# Circulating long noncoding RNAs as potential biomarkers for stomach cancer: a systematic review and meta-analysis

**DOI:** 10.1186/s12957-021-02194-6

**Published:** 2021-03-26

**Authors:** Fang Cao, Yongwei Hu, Zaichang Chen, Wei Han, Weijie Lu, Jianhao Xu, Houzhong Ding, Xiaojun Shen

**Affiliations:** 1 Department of General surgery, Kunshan First People’s Hospital Affiliated to Jiangsu University, Kunshan, Jiangsu China; 2 Department of Pathology, Kunshan First People’s Hospital Affiliated to Jiangsu University, Kunshan, Jiangsu China

**Keywords:** Stomach cancer, Circulating lncRNAs, Diagnosis, Meta-analysis

## Abstract

**Background:**

Recent researches have suggested that long noncoding RNA (lncRNA) is involved in the tumorigenesis and development of stomach cancer (SC). This meta-analysis aimed to identify the diagnostic performance of circulating lncRNAs in SC.

**Methods:**

All relevant studies were systematically searched through PubMed, Web of Science, Cochrane Library, and EMBASE databases. The diagnostic values of lncRNAs were mainly assessed by pooled sensitivity, specificity, and summary receiver operating characteristic area under the curve (SROC AUC). Meta-DiSc 1.4, Review Manager 5.3, and STATA 12.0 were used for statistical analysis. The protocol for this systematic review was registered on INPLASY (INPLASY202120079) and is available in full on the inplasy.com (10.37766/inplasy2021.2.0079).

**Results:**

A total of 42 eligible studies were included in this meta-analysis. The pooled sensitivity, specificity, and SROC AUC were 0.78 (95%CI 0.75–0.81), 0.75 (95%CI 0.71–0.78), and 0.83 (95%CI 0.80–0.86), respectively, suggesting that the lncRNAs test had a high accuracy for the diagnosis of SC. Obvious heterogeneity might come from the type of lncRNA through subgroup and meta-regression analysis. Fagan diagram shows the clinical value of lncRNAs test in SC.

**Conclusions:**

Abnormal expression of circulating lncRNAs exhibits a high efficacy for diagnosing SC, which is promising in clinical application.

**Supplementary Information:**

The online version contains supplementary material available at 10.1186/s12957-021-02194-6.

## Background

Based on 2018 global cancer data, stomach cancer (SC) is the 5th most common neoplasm and the 3rd most deadly cancer, causing an estimated 783,000 deaths in 2018 [1]. Studies have shown that SC patients are often diagnosed at later stages due to the absence of typical early signs [2]. As a result, the overall survival in patients with advanced SC is poor; the 5-year survival rate ranges from approximately 10 to 30% [3]. The prognosis of SC is highly dependent on the timing of the diagnosis [4]. Blood-based cancer biomarkers are ideal for screening and early detection due to their convenience and low invasiveness. However, the low sensitivity and specificity of conventional blood biomarkers limit their application, such as carcinoembryonic antigen and carbohydrate antigen 19-9 [5]. Although considerable effort has been devoted to identifying the underlying mechanism of SC, the identification of new diagnostic markers for SC is still a considerable challenge.

In recent years, the regulation of gene expression by noncoding RNAs has been studied thoroughly. Long noncoding RNAs (lncRNAs) are RNA molecules greater than 200 nucleotides that modulate gene expression at the levels of transcription, posttranscription, and translation, but are not able to encode proteins [6]. An increasing body of evidence has suggested that lncRNAs play a major role during the processes of tumorigenesis and development, which may offer new ideas for the early diagnosis of SC. For instance, for distinguishing SC patients from normal subjects, the lncRNAs PCGEM1 and LOC80054 have higher area under the curve (AUC) values than other conventional tumor markers (AFP, CEA, CA12-5, CA19-9, and CA72-4) [7, 8]. Similarly, lncRNAs can also be detected in the blood, and circulating noncoding RNAs have become a new source of noninvasive cancer biomarkers [9], which can serve as new diagnostic biomarkers for SC.

However, considering the small sample size and limitations of the research design, there is insufficient evidence to confirm the diagnostic accuracy of circulating lncRNAs in SC patients. To address this shortcoming, a comprehensive systematic review and meta-analysis was conducted to explore the diagnostic accuracy of circulating lncRNAs in SC.

## Methods

### Search strategy

This meta-analysis was conducted in accordance with the Preferred Reporting Items for Systematic Reviews and Meta-Analyses guidelines [10]. The PubMed, Web of Science, Cochrane Library, and Embase databases were systematically searched for potentially relevant articles, which were independently screened by two authors (Cao F and Xu J). The reference lists of relevant meta-analyses and reviews were also searched to identify articles that were not included in the initial search. In addition, relevant articles in scientific congresses and conferences were reviewed. The search strategy and Participant, Index test, Comparison, Outcome, and Study (PICOS) design strategy are shown in Table 1. The publication search was updated regularly until July 9, 2020.

**Table 1:** Systematic search strategy (PICOS strategy)

Search strategy	
Participant	#1 (Stomach Neoplasms[MeSH Terms] OR”Neoplasm, Stomach"OR"Stomach Neoplasm"OR"Neoplasms, Stomach"OR"Stomach Neoplasms"OR"Gastric Neoplasms"OR"Gastric Neoplasm"OR"Neoplasm, Gastric"OR"Neoplasms, Gastric"OR"Cancer of Stomach"OR"Stomach Cancers"OR"Gastric Cancer"OR"Cancer, Gastric"OR"Cancers, Gastric"OR"Gastric Cancers"OR"Stomach Cancer"OR"Cancer, Stomach"OR"Cancers, Stomach"OR"Cancer of the Stomach"OR"Gastric Cancer, Familial Diffuse”)
Index test	#2 (RNA, Long Noncoding[MeSH Terms]OR“RNA, Long Noncoding”OR“Noncoding RNA, Long”OR”lncRNA”OR”Long ncRNA”OR”ncRNA, Long”OR”RNA, Long Non-Translated”OR”Long Non-Translated RNA”OR”Non-Translated RNA, Long”OR”RNA, Long Non Translated”OR”Long Non-Coding RNA”OR”Long Non Coding RNA”OR”Non-Coding RNA, Long”OR”RNA, Long Non-Coding”OR”Long Non-Protein-Coding RNA”OR”Long Non Protein Coding RNA”OR”Non-Protein-Coding RNA, Long”OR”RNA, Long Non-Protein-Coding”OR”Long Noncoding RNA”OR”RNA, Long Untranslated”OR”Long Untranslated RNA”OR”Untranslated RNA, Long”OR”Long ncRNAs”OR”ncRNAs, Long”OR”Long Intergenic Non-Protein Coding RNA”OR”Long Intergenic Non Protein Coding RNA”OR”LincRNAs”OR”LINC RNA”)
Comparison	None
Outcome	#3 (Biomarkers[MeSH Terms]OR“Biomarkers”OR”Biomarker”OR”Markers, Biological”OR“Biologic Markers”OR“Markers, Biologic”OR“Biologic Marker”OR“Marker, Biologic"OR“Marker, Biological”OR“Biological Marker”OR“Biological Markers”OR“Markers, Laboratory”OR“Laboratory Markers”OR“Laboratory Marker”OR“Marker, Laboratory”OR“Serum Markers”OR“Markers, Serum”OR“Marker, Serum”OR“Serum Marker”OR“Plasma Markers”OR“Markers, Plasma”OR“Marker, Plasma”OR“Plasma Marker”OR“Markers, Clinical”OR“Clinical Markers”OR“Clinical Marker”OR“Marker, Clinical”) #4 (Sensitivity and Specificity[MeSH Terms]OR“Sensitivity and Specificity”OR“Specificity and Sensitivity”OR”Sensitivity”OR”Specificity”) OR (Diagnosis[MeSH Terms]OR“Diagnosis”OR“Diagnose”OR”Diagnostic”OR“Diagnoses”OR”Diagnoses and Examinations”OR”Examinations and Diagnoses”OR”Postmortem Diagnosis”OR”Diagnoses, Postmortem”OR”Diagnosis, Postmortem”OR”Postmortem Diagnoses”OR”Antemortem Diagnosis”OR”Antemortem Diagnoses”OR”Diagnoses, Antemortem”OR”Diagnosis, Antemortem”)
Study design	None
Search	#1 AND #2 AND (#3 OR #4)
Database search	
Language	No restriction
Electronic databases	PubMed, Web of Science, Cochrane Library, and Embase databases

### Selection criteria

The following inclusion criteria were used:

(i) The expression of lncRNAs was determined in plasma or serum by quantitative reverse transcription-polymerase chain reaction or other molecular techniques;
(ii) Studies evaluated the diagnosis value of lncRNA for SC;
(iii) Sufficient data to determine false negatives, true negatives, false positives, and true positives.

The exclusion criteria were as follows:

(i) Duplicate publications;
(ii) Meta-analysis, correspondence, single case reports, review articles, and animal model studies.

### Data extraction

The two authors (Cao F and Xu J) reviewed the full texts and independently extracted data from all included studies. The following data were extracted: first author, year of publication, race of participants, pathological type of experimental group/control group, sample size, specimen type, lncRNA type, dysregulated state of lncRNAs, sensitivity, and specificity.

### Quality assessment

Two authors (Xu J and Cao F) independently evaluated the quality of each diagnostic study. The methodological quality and applicability of the included studies were examined using the Quality Assessment of Diagnostic Accuracy Studies 2 (QUADAS-2) [11] tool in using Review Manager software version 5.3. The QUADAS-2 tool is used to assess the quality of diagnostic accuracy studies [11]. The QUADAS-2 tool contains 4 main areas: process and timing, index testing, reference standards, and patient selection. The risk of prejudice and apprehension was classified as “low,” “high,” or “unclear.” The differences were resolved through discussions among all the researchers.

Details of the protocol for this systematic review were registered on INPLASY (INPLASY202120079) and are available in full on the inplasy.com (10.37766/inplasy2021.2.0079). This study is presented in accordance with the Preferred Reporting Items for Systematic Reviews and Meta-Analyses (PRISMA) Statement.

### Statistical analysis

Meta-analyses were performed using Meta-DiSc 1.4 (Romany Cajal Hospital, Madrid, Spain) [12], Review Manager 5.3 (Cochrane Collaboration, Oxford, England), and STATA 12.0 (Stata Corp LP, TX, USA).

For a meta-analysis of diagnostic accuracy, the sensitivity, specificity, negative likelihood ratio, positive likelihood ratio, diagnostic odds ratio, and the corresponding 95% CIs were used to determine the diagnostic value of lncRNAs. To quantitatively assess the accuracy of diagnosis, the area under the curves (AUCs) of summary receiver operating characteristic curves (SROCs) were determined. The SROC curve method is a meta-analysis of multiple different experiments of a certain detection index. According to the weight of their odds ratio, the diagnostic accuracy is comprehensively evaluated by fitting the SROC curve [13]. The hierarchical summary receiver operating characteristic (HSROC) model proposed by Rutter and Gatsonis in 2001 represents a general framework for the meta-analysis of diagnostic test studies that allows different parameters to be defined as random effects [14]. A HSROC model was adopted to extend the fixed-effects SROC model and evaluate the accuracy of multiple diagnostic tests.

The heterogeneity tests were carried out by the *Q* test and *I*^2^ statistics. *P* values of < 0.05 were regarded as statistically significant. An *I*^2^ value > 50% and a *P* value < 0.05 indicated significant heterogeneity between the included studies, and a random effects model was applied. Otherwise, if there was no obvious heterogeneity, the fixed effects model was applied to evaluate the aggregated results. The heterogeneity induced by the threshold effect was evaluated by the ROC plane. Galbraith Star charts and bivariate boxplots were employed to estimate the degree of heterogeneity. Subgroup analysis and meta-regression were used to assess the source of heterogeneity. Subgroup results were examined one at a time.

Sensitivity analysis was used to determine the stability of the results. Potential publication bias was examined by Deeks’ funnel plot. A *P* value of > 0.1 indicates that there is no publication bias. Fagan’s nomogram was applied to judge the clinical value of lncRNAs as a diagnostic method.

## Results

### Literature searching and study screening

**Fig. 1:**
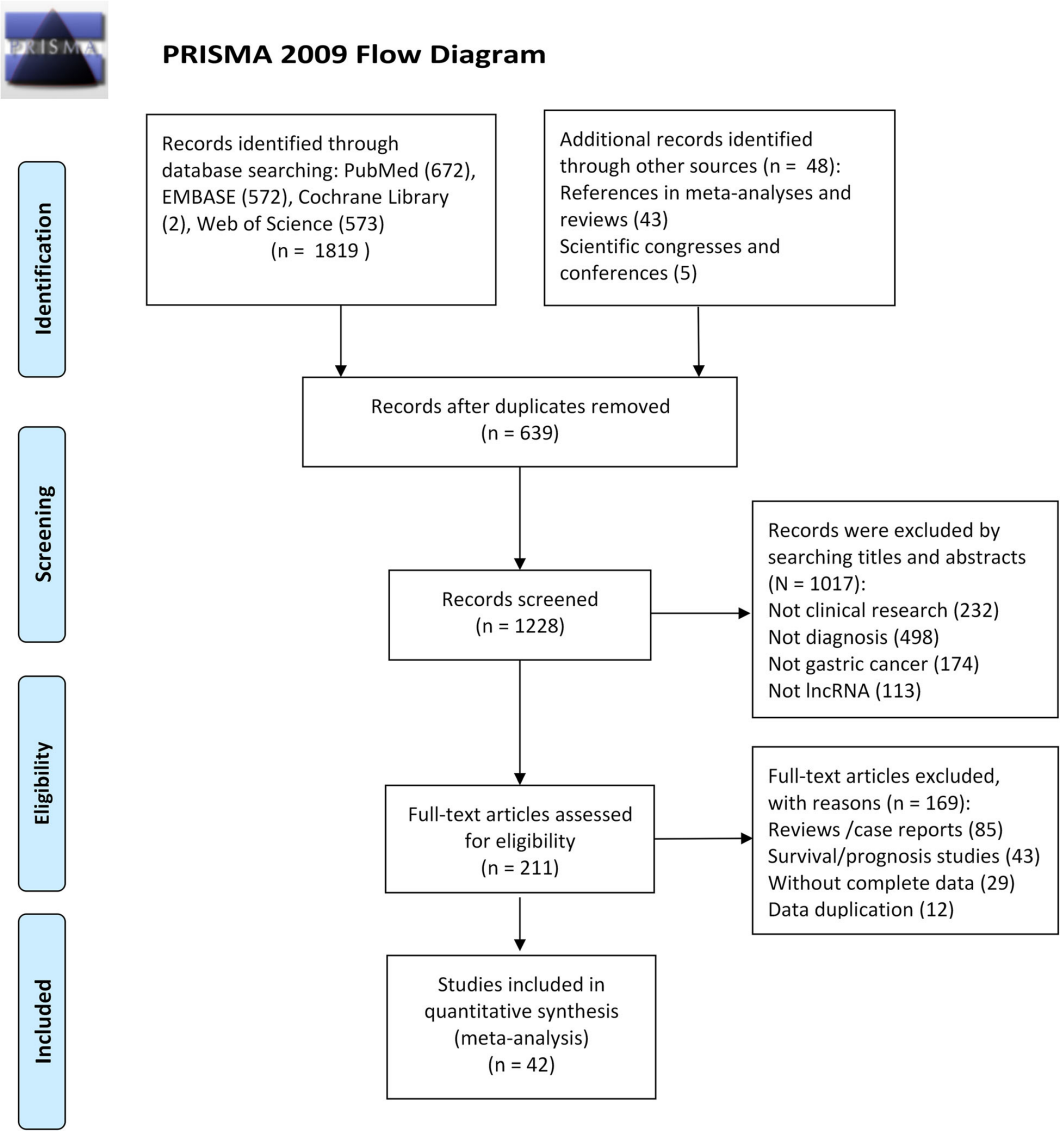
Flow chart of the study selection process

In total, 1867 articles were obtained from the four databases. After eliminating 639 duplicate articles, 1228 studies were further screened. After screening the titles, abstracts, and full texts, 42 eligible studies [2, 7, 15–54] were finally included based on the selection criteria (Fig. 1).

### Quality evaluation and main characteristics of the eligible studies

**Fig. 2:**
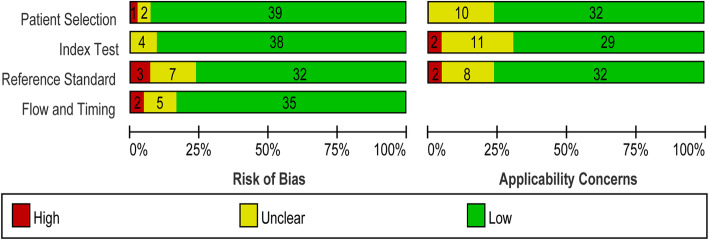
Quality assessment of eligible studies for diagnostic meta-analysis

The diagnostic meta-analysis analyzed 42 eligible studies [2, 7, 15–54] published between 2013 and 2020. Thirty-seven studies detected lncRNA expression in Asian population, while 5 studies detected lncRNA expression in Caucasian populations. Sample types included plasma, serum, and plasma/serum exosomes. All SC patients were pathologically confirmed, and the control groups consisted of healthy donor individuals and benign stomach disease patients. A total of 49 different lncRNAs were examined across all included studies; most of the lncRNAs were upregulated in SC (Table 2). The quality assessment is shown in Fig. 2.

**Table 2:** Main characteristics of eligible studies for diagnosis

First author, year	Race	Pathologictype (E/C)	Sample size (E/C)	Specimen	lncRNA	State	Sen	Spe	TP	FP	FN	TN	QUADAS-2	(Refs)
Liu, 2019	Asian	GC/HD	89/73	Serum	FEZF1-AS1	Up	75.3%	65.8%	67	25	22	48	5	[15]
					AFAP1-AS1	Up	76.4%	56.2%	68	32	21	41		
Yoruke, 2018	Caucasian	GC/non-GC	40/42	Plasma	H19	Up	87.2%	38.1%	35	26	5	16	6	[16]
Liu, 2019	Asian	GC/HD	100/100	Serum	MALAT1	Up	85.8%	74.5%	86	26	14	75	4	[17]
Hashad, 2016	Caucasian	GC/HD	32/30	Plasma	H19	Up	68.8%	56.7%	22	13	10	17	5	[18]
Li, 2014	Asian	GC/HD	79/81	Plasma exosome	LINC00152	Down	48.1%	85.2%	38	12	41	69	6	[19]
Liu, 2014	Asian	GC/HD	83/80	Plasma	FER1L4	Up	67.2%	80.3%	56	16	27	64	7	[20]
Liu, 2018	Asian	GC/HD	50/50	Plasma	CTC-501O10.1	Up	90.0%	51.0%	45	25	5	26	6	[21]
					AC100830.4	Up	84.0%	58.0%	42	21	8	29		
					RP11-210K20.5	Up	89.0%	55.0%	45	23	6	28		
Lu, 2017	Asian	EGC/HD	76/76	Serum	XIST	Up	84.6%	59.0%	64	31	12	45	5	[22]
					BCYRN1	Up	67.9%	85.9%	52	11	24	65		
					RRP1B	Down	85.9%	56.4%	65	33	11	43		
					TDRG1	Down	73.1%	60.3%	56	30	20	46		
Mohamed, 2019	Caucasian	GC/HD	35/25	Serum	H19	Up	90.9%	100.0%	32	0	3	25	5	[23]
Piao, 2020	Asian	GC/HD	281/80	Plasma exosome	CEBPA-AS1	Up	74.0%	88.0%	208	10	73	70	6	[24]
Zhou, 2016	Asian	GC/HD	77/60	Plasma	ZFAS1	Up	76.6%	63.9%	59	22	18	38	5	[25]
Cai, 2019	Asian	GC/HD	63/29	Serum exosome	PCSK2-2:1	Up	84.0%	86.5%	53	4	10	25	6	[26]
Zhou, 2015	Asian	GC/HD	90/90	Plasma	H19	Up	82.9%	72.9%	75	24	15	66	7	[27]
Elsayed, 2018	Caucasian	GC/HD	50/50	Plasma	HOTAIR	Up	86.0%	94.0%	43	3	7	47	4	[28]
Xian, 2018	Asian	GC/HD	50/50	Plasma	HULC	Up	58.0%	80.0%	29	10	21	40	5	[29]
					ZNFX1-AS1	Up	84.0%	68.0%	42	16	8	34		
Feng, 2019	Asian	GC/HD	107/87	Serum	B3GALT5-AS1	Up	64.5%	87.4%	69	11	38	76	5	[30]
Fu, 2017	Asian	GC/HD	72/72	Serum	LINC00978	Up	80.0%	70.0%	58	22	14	50	5	[31]
Gao, 2015	Asian	GC/HD	20/20	Plasma	UCA1	Up	85.0%	96.3%	17	1	3	19	6	[32]
					PVT1	Down	70.8%	91.3%	14	2	6	18		
Ghaedi, 2018	Asian	GC/HD	62/40	Plasma	H19	Up	74.2%	90.0%	46	4	16	36	6	[33]
					MEG3	Down	77.4%	52.5%	48	19	14	21		
Guo, 2020	Asian	EGC/HD	217/219	Plasma exosome	GC1	Up	97.0%	83.0%	210	37	7	182	5	[34]
Arita, 2013	Asian	GC/HD	43/34	Plasma	H19	Up	74.0%	58.0%	32	14	11	20	6	[35]
Ji, 2019	Asian	GC/HD	168/74	Plasma	LINC00086	Down	72.6%	83.8%	122	12	46	62	7	[36]
Jiang, 2019	Asian	GC/HD	317/100	Plasma	PCGEM1	Up	72.9%	88.9%	231	11	86	89	7	[7]
Lin, 2018	Asian	GC/HD	51/60	Plasma exosome	UEGC1	Up	88.0%	82.0%	45	11	6	49	5	[37]
					UEGC2	Up	89.0%	58.0%	45	25	6	35		
Pan, 2017	Asian	GC/HD	60/37	Serum exosome	ZFAS1	Up	71.7%	75.7%	43	9	17	28	5	[38]
Jin, 2016	Asian	GC/HD	173/110	Serum	HULC	Up	82.0%	83.6%	142	18	31	92	6	[39]
Zhang, 2018	Asian	GC/HD	57/29	Serum exosome	UFC1	Up	78.0%	80.0%	44	6	13	23	5	[2]
Zhao, 2018	Asian	GC/HD	126/120	Serum exosome	HOTTIP	Up	69.8%	85.0%	88	18	38	102	4	[40]
Burock, 2015	Caucasian	GC/non-GC	76/54	Plasma	MACC1	Up	68.0%	89.0%	52	6	24	48	5	[41]
Ke, 2017	Asian	GC/HD	51/53	Plasma	INHBAAS1	Down	92.7%	74.5%	47	14	4	39	6	[42]
					MIR4435-2HG	Down	90.2%	74.5%	46	14	5	39		
					CEBPA-AS1	Down	78.0%	76.6%	40	12	11	41		
					UCA1	Down	73.2%	82.3%	37	9	14	44		
					AK001058	Down	95.1%	72.3%	49	15	2	38		
			47/52	Plasma	INHBAAS1	Down	82.7%	59.6%	39	21	8	31		
					MIR4435-2HG	Down	65.4%	87.2%	31	7	16	45		
					CEBPA-AS1	Down	96.2%	57.4%	45	22	2	30		
					AK001058	Down	76.9%	92.3%	36	4	11	48		
Liu, 2019	Asian	GC/HD	94/40	Serum	HOXA11-AS	Up	78.7%	97.8%	74	1	20	39	7	[43]
Shan, 2019	Asian	GC/HD	117/100	Serum	UCA1	Up	93.2%	78.6%	109	21	8	79	6	[44]
Shao, 2016	Asian	GC/HD	83/90	Plasma	RMRP	Down	59.1%	67.8%	49	29	34	61	5	[45]
Yang, 2019	Asian	GC/HD	109/106	Plasma	FOXD2-AS1	Up	83.0%	50.0%	90	53	19	53	5	[46]
					PANDAR	Up	85.0%	63.0%	93	39	16	67		
					SMARCC2	Up	90.0%	55.0%	98	48	11	58		
Xu, 2020	Asian	GC/HD	109/50	Serum	MIAT	Up	81.5%	87.5%	89	6	20	44	5	[47]
Xu, 2018	Asian	GC/HD	34/34	Plasma	DGCR5	Down	58.0%	87.0%	20	4	14	30	6	[48]
Xu, 2019	Asian	GC/HD	45/45	Plasma	LINC01225	Up	50.0%	90.0%	23	5	23	41	4	[49]
Yang, 2016	Asian	GC/HD+GS	133/152	Serum	H19	Up	65.0%	53.0%	86	71	47	81	7	[50]
					LINC00152	Up	40.0%	72.0%	53	43	80	109		
Zheng, 2020	Asian	GC/HD	60/60	Plasma	SLC2A12-10:1	Up	68.0%	75.0%	41	15	19	45	5	[51]
Zheng, 2018	Asian	GC/HD	241/228	Plasma	FAM49B-AS	Up	58.0%	60.0%	140	91	101	137	6	[52]
					GUSBP11	Up	46.0%	75.0%	111	57	130	171		
					CTDHUT	Up	73.0%	65.0%	176	80	65	148		
Zhou, 2020	Asian	GC/GS+GA+GD	200/278	Serum	C5orf66-AS1	Down	77.5%	53.6%	155	129	45	149	6	[53]
Tan, 2016	Asian	GC/HD	117/80	Plasma	GACAT2	Down	87.2%	28.2%	102	57	15	23	5	[54]

*E/C* experimental group/control group, *GC* gastric cancer, *EGC* early gastric cancer, *HD* healthy donor individuals, *GS* superficial gastritis, *GA* atrophic gastritis, *GD* gastric dysplasia, *SEN* sensitivity, *SPE* specificity, *TP* true positive, *FP* false positive, *FN* false negative, *TN* true negative, *QUADAS-2* Quality Assessment of Diagnostic Accuracy Studies 2

### Diagnostic accuracy of lncRNA

**Fig. 3:**
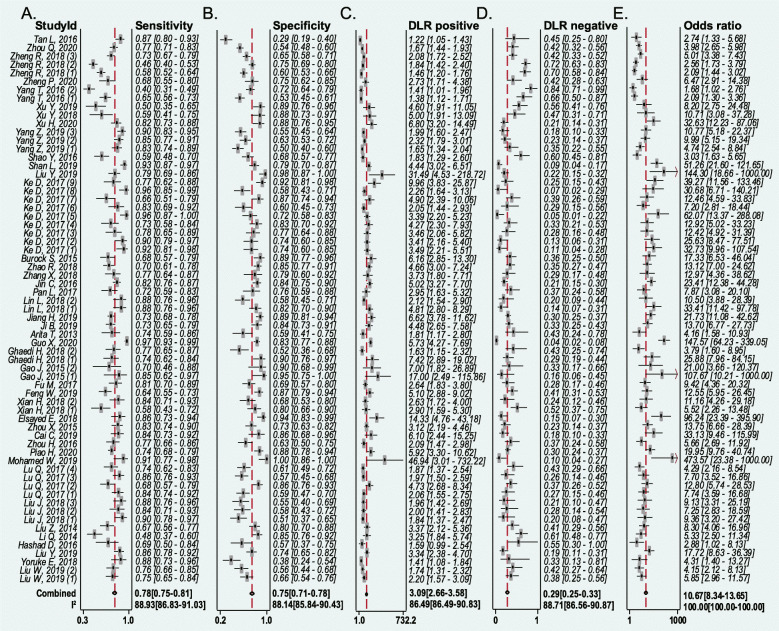
Forest plots of the diagnostic value for lncRNAs test in detecting SC. **a** Sensitivity, **b** specificity, **c** positive likelihood ratio, **d** negative likelihood ratio, **e** diagnostic odds ratio

**Fig. 4:**
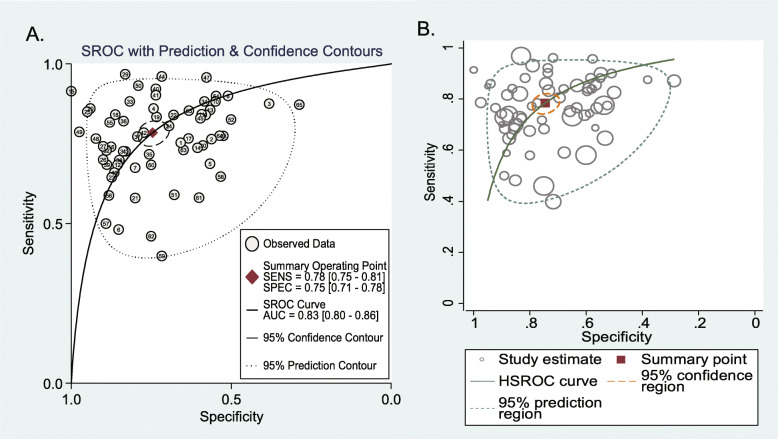
SROC curve of lncRNAs test in detecting SC. **a** SROC curve and **b** HSROC model.

A total of 42 eligible diagnostic studies were meta-analyzed. As illustrated in Fig. 3, the pooled sensitivity, specificity, positive likelihood ratio, negative likelihood ratio, and diagnostic odds ratio were 0.78 (95% CI 0.75–0.81), 0.75 (95% CI 0.71–0.78), 3.09 (95% CI 2.66–3.58), 0.29 (95% CI 0.25–0.33), and 10.67 (95% CI 8.34–13.65), respectively. As demonstrated in Fig. 4a, the AUC value of the SROC was 0.83 (95% CI 0.80–0.86). The SROC results were further evaluated through the HSROC model. As shown in Fig. 4b, the *β* estimate was 0.11 (95% CI −0.19−0.40) and the corresponding *P* value was 0.485. The lambda estimate was 2.38 (95% CI 2.13–2.63).

### Heterogeneity analysis

As illustrated in Fig. 3, obvious heterogeneity was found in the pooled sensitivity (*I*^2^ = 88.93%, *P* < 0.01), specificity (*I*^2^ = 88.14%, *P* < 0.01), positive likelihood ratio (*I*^2^ = 88.49%, *P* < 0.01), negative likelihood ratio (*I*^2^ = 88.71%, *P* < 0.01), and diagnostic odds ratio (*I*^2^ = 100.00%, *P* < 0.01).

**Fig. 5:**
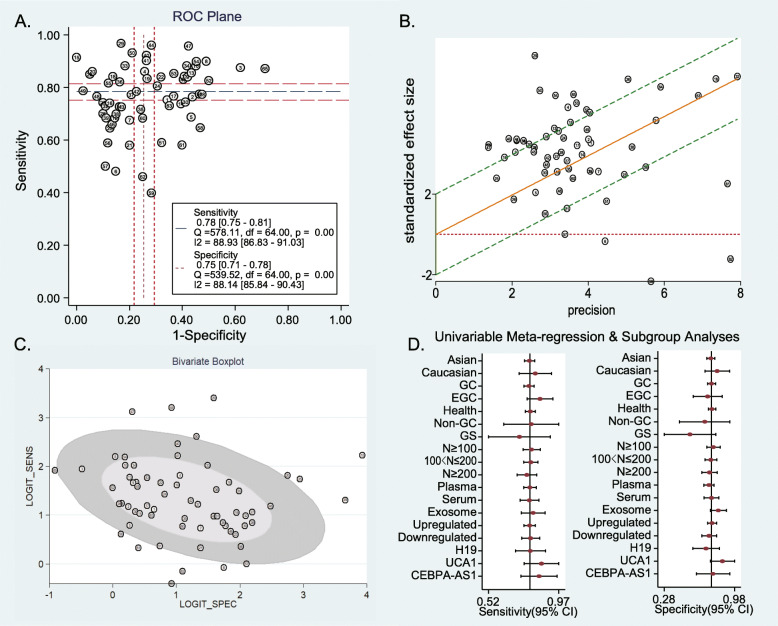
Heterogeneity analysis of diagnostic tests. **a** ROC plane of the pooled studies. **b** Galbraith star charts of the pooled studies. **c** Bivariate boxplots of the pooled studies. **d** Subgroup and meta-regression analysis for heterogeneity of the pooled studies

A nontypical shoulder arm appearance was observed in the ROC plane (Fig. 5a). Twenty out of the 63 studies of the Galbraith star chart and 10 out of 42 studies of the bivariate box plot fell outside the 95% CI (Fig. 5b, c). Figure 5d shows the meta-regression forest map. All studies were grouped according to race, pathological types of experimental groups, pathological types of control groups, sample size, specimen type, dysregulated state of lncRNAs, and lncRNA types. Table 3 shows the changes in sensitivity, specificity, and *I*^2^ values after meta-regression and subgroup analysis.

**Table 3:** Subgroup analysis of the diagnostic efficacy of lncRNA in stomach cancer

Group	Subgroup	No. of studies	No. of patients	Sensitivity	Heterogeneity (*I*^2^, *P* value)	Specificity	Heterogeneity (*I*^2^, *P* value)	AUC	Meta-regression (*P* value)
Overall		42	7524	0.78 [0.75, 0.81]	88.93%; <0.001	0.75 [0.71, 0.78]	88.14%; <0.001	0.83 [0.80–0.86]	
Race	Asian	60	7090	0.78 [0.75, 0.81]	89.38%; <0.001	0.74 [0.70, 0.77]	87.51%; <0.001	0.83 [0.79–0.86]	0.48
	Caucasian	5	434	0.81 [0.70, 0.89]	75.16%; <0.001	0.86 [0.52, 0.97]	94.22%; <0.001	0.87 [0.84–0.90]	0.48
Pathologic types (E)	GC	60	6936	0.78 [0.74, 0.81]	88.19%; <0.001	0.75 [0.71, 0.79]	88.35%; <0.001	0.83 [0.80–0.86]	0.38
	EGC	5	588	0.85 [0.72, 0.93]	91.39%; <0.001	0.71 [0.58, 0.81]	90.27%; <0.001	0.84 [0.80–0.87]	0.38
Pathologic types (C)	health	61	6549	0.79 [0.75, 0.82]	89.32%; <0.001	0.75 [0.71, 0.79]	86.53%; <0.001	0.84 [0.80–0.87]	0.15
	non-GC	2	212	–	–	–	–	–	0.86
	GS	2	763	–	–	–	–	–	0.10
Sample size	N≤100	21	1153	0.79 [0.74, 0.84]	74.67%; <0.001	0.77 [0.69, 0.84]	84.82%; <0.001	0.85 [0.82–0.88]	0.62
	100<N≤200	28	2722	0.79 [0.74, 0.82]	79.14%; <0.001	0.74 [0.68, 0.79]	85.75%; <0.001	0.83 [0.80–0.86]	0.95
	N>200	16	3649	0.77 [0.68, 0.84]	95.41%; <0.001	0.73 [0.65, 0.79]	92.57%; <0.001	0.81 [0.77–0.84]	0.58
Specimen	Plasma	23	3467	0.78 [0.74, 0.82]	87.25%; <0.001	0.72 [0.67, 0.77]	86.87%; <0.001	0.82 [0.79–0.85]	0.34
	Serum	12	2468	0.78 [0.72, 0.83]	90.56%; <0.001	0.75 [0.67, 0.82]	90.37%; <0.001	0.84 [0.80–0.87]	0.99
	Exosome	8	1589	0.81 [0.70, 0.89]	92.18%; <0.001	0.81 [0.76, 0.86]	69.40%; <0.001	0.87 [0.84–0.90]	0.17
Dysregulated state	Upregulated	46	6003	0.78 [0.74, 0.82]	90.19%; <0.001	0.75 [0.71, 0.80]	87.40%; <0.001	0.84 [0.80–0.87]	0.78
	Downregulated	19	1521	0.79 [0.72, 0.84]	84.54%; <0.001	0.72 [0.64, 0.79]	89.12%; <0.001	0.82 [0.79–0.86]	0.78
lncRNA	H19	7	848	0.78 [0.70, 0.84]	75.98%; <0.001	0.72 [0.49, 0.88]	89.62%; <0.001	0.82 [0.78–0.85]	0.69
	UCA1	3	361	0.87 [0.81, 0.91]	83.60%; 0.02	0.82 [0.76, 0.88]	45.20%; 0.161	0.92 [0.84–0.99]	0.13
	CEBPA-AS1	3	564	0.77 [0.73, 0.81]	86.00%; 0.001	0.76 [0.69, 0.82]	86.80%; 0.001	0.88 [0.84–0.92]	0.63

*E/C* experimental group/control group, *GC* gastric cancer, *EGC* early gastric cancer, *HD* healthy donor individuals, *GS* superficial gastritis, *AUC* area under the curve

### Sensitivity analysis and publication bias

First, sensitivity analysis was carried out to determine the stability of our results. The removal of individual studies exhibited no noticeable changes in pooled results (Additional file 1: Supplementary Figure 1, Fig. S1A). The *P* value of Deeks’ funnel plot asymmetry test was 0.12 (Additional file 1: Supplementary Figure 1, Fig. S1B).

### Clinical values of lncRNAs for SC diagnosis

**Fig. 6:**
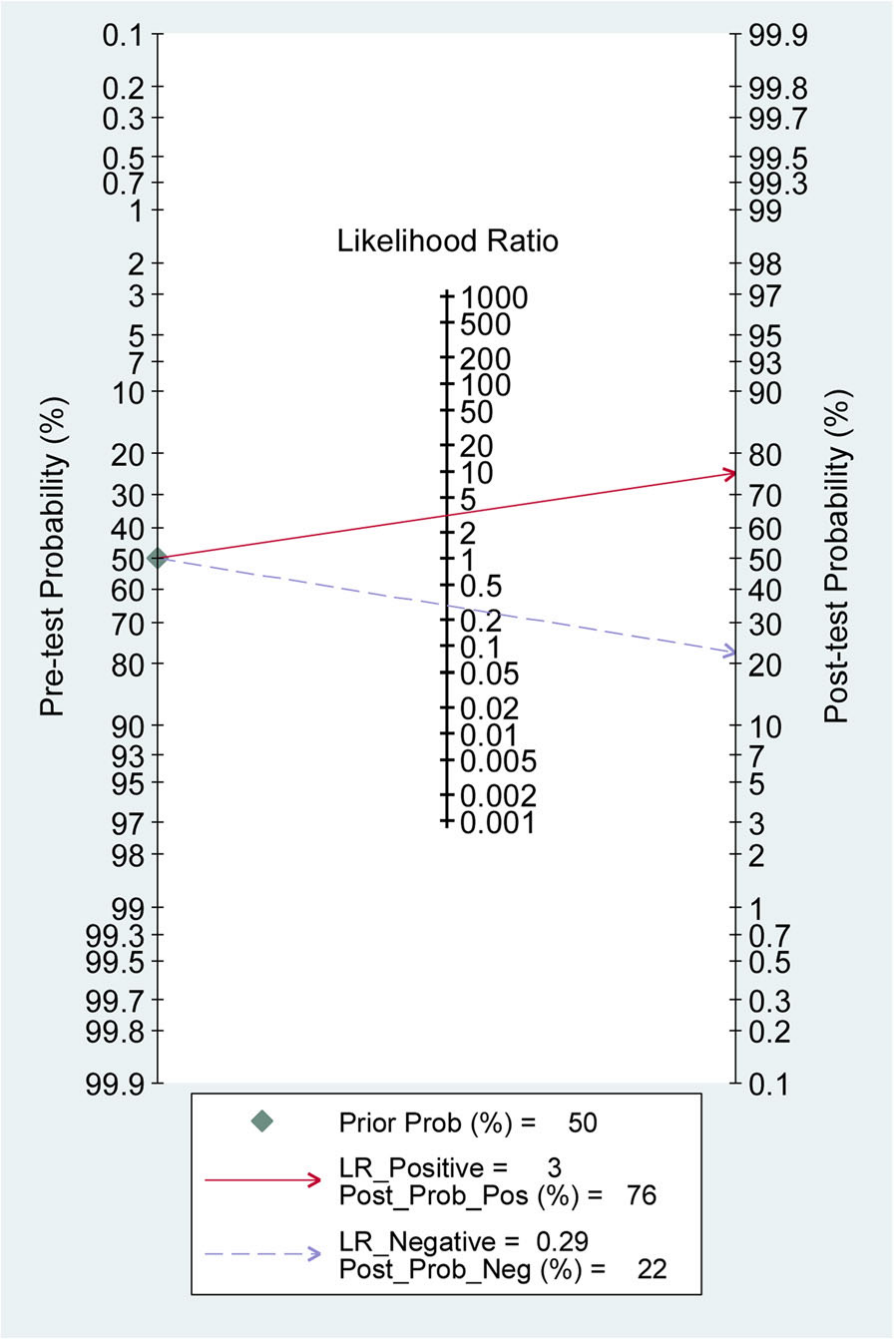
Fagan’s nomogram of lncRNAs test in detecting SC

As shown in Fig. 6, Fagan’s nomogram revealed that if the patient had a positive lncRNA test result, the actual probability of suffering from SC was 76%, while the probability was 22% if a negative test result was obtained.

## Discussion

In recent years, lncRNAs have been recognized as potential diagnostic biomarkers for different cancers [55]. As a diagnostic biomarker for cancer, lncRNAs have the following special advantages. First, the abundance of lncRNAs is relatively high. In the human genome, the number of lncRNAs is four times greater than that of coding RNAs [56]. Second, lncRNAs are highly expressed in the plasma, tissue, and exosomes of cancer cases [57]. Third, lncRNAs have complex biological functions and are closely related to tumorigenesis and development. Therefore, lncRNAs may be promising biomarkers for the early detection and prognosis of various cancers [58].

In the present meta-analysis, a total of 42 eligible studies were screened. The aggregated results of sensitivity, specificity, positive likelihood ratio, negative likelihood ratio, diagnostic odds ratio, and SROC AUC indicated that the abnormal expression of circulating lncRNAs exhibits a high accuracy for the diagnosis of SC. The βestimate in the HSROC model indicated that the SROC is symmetrical. Meanwhile, the estimate of lambda reflected the diagnostic accuracy of lncRNAs. Sensitivity analysis verified the stability of the results, and the Deeks funnel chart asymmetry test showed that there was no obvious publication bias. The Fagan diagram also shows its advantages in clinical application, which was mainly due to its moderately high positive and negative predictive value.

For the obvious heterogeneity in the pooled estimates, many analyses have been applied to explore the source of heterogeneity. The ROC plane suggests the absence of a threshold effect, while the Galbraith star charts and bivariate boxplots suggest heterogeneity between studies. Meta-regression and subgroup analysis showed that the heterogeneity might come from the type of lncRNA: when lncRNA UCA1 was used as the grouping condition, the *I*^2^ of sensitivity was reduced to 83.60%, and the *I*^2^ of specificity was reduced to 45.20% (*P* = 0.161). In addition, the diagnostic value of lncRNA UCA1 was above average (AUC: 0.92 (95% CI 0.84–0.99) versus 0.83 (95% CI 0.80–0.86)). There was no evidence that race, pathological types of experimental groups, pathological types of control groups, sample size, specimen type, and dysregulated state of lncRNAs significantly affected the pooled results.

Although meta-analysis of lncRNAs in the diagnosis of SC has been reported before [59, 60], most of them focus on lncRNAs in SC tissues. Although lncRNAs in tissue also have high diagnostic accuracy (AUC= 0.755 [59]; 0.80 [60]), their clinical application value is limited for the following reasons: first, the diagnosis of SC after surgery depends on the pathological morphology and immunohistochemical analysis, and the auxiliary role of lncRNAs is optional; second, in regard to endoscopic biopsy specimens, the diagnosis of SC still depends on the pathological morphology, and no extra tumor tissue can be used to extract lncRNAs. In contrast, circulating lncRNAs are ideal biomarkers due to their convenience and low invasiveness. Therefore, the present study on the application of circulating lncRNAs in the diagnosis of SC has greater clinical significance.

Nevertheless, this meta-analysis possessed some limitations. First, this systematic review and meta-analysis lacks eligible non-Asian studies. Second, almost every study focuses on different lncRNAs, and it was difficult to perform subgroup analysis based on lncRNA types to explain the possible sources of heterogeneity. Third, obvious heterogeneity was found in the included studies. Although diagnostic meta-analysis suggested that the type of lncRNA was a source of heterogeneity through meta-regression and subgroup analysis, the heterogeneity of sensitivity and specificity were still high in each subgroup.

## Conclusions

In conclusion, the findings of the diagnostic meta-analysis provide evidence that circulating lncRNA tests exhibit a high accuracy for diagnosing SC, which is promising in clinical application due to their high positive and negative predictive value. This study provides an important reference value for the application of circulating lncRNAs as biomarkers for the early diagnosis of SC. Due to potential limitations, further investigations are warranted to verify the diagnostic role of circulating lncRNAs in SC.

## Supplementary Information

**Additional file 1 **: **Fig S1**. Sensitivity analysis and publication bias. (A) Sensitivity analysis of the pooled studies. (B) Deeks’ funnel plot of the pooled studies.

## Data Availability

All relevant data are within the paper and its additional files.

## Abbreviations

LncRNA: Long noncoding RNA
SC: Stomach cancer
SROC: Summary receiver operating characteristic
AUC: Area under the curve
CI: Confidence interval
CA-153: Cancer antigen 153
CEA: Carcinoembryonic antigen
QUADAS-2: Quality Assessment of Diagnostic Accuracy Studies 2
HSROC: Hierarchical summary receiver operator characteristic
