# Supplementary material for: Circulating long noncoding RNAs as potential biomarkers for stomach cancer: a systematic review and meta-analysis
Source: World J Surg Oncol. 2021 Mar 26;19:89. doi: 10.1186/s12957-021-02194-6 (PMC8004465; doi:10.1186/s12957-021-02194-6)
Supplement: Supplementary file 1 — Additional file 1 : Fig S1. Sensitivity analysis and publication bias. (A) Sensitivity analysis of the pooled studies. (B) Deeks’ funnel plot of the pooled studies. [file 12957_2021_2194_MOESM1_ESM.zip › Additional file 1.docx]

Additional file 1: Supplementary Figure 1

**
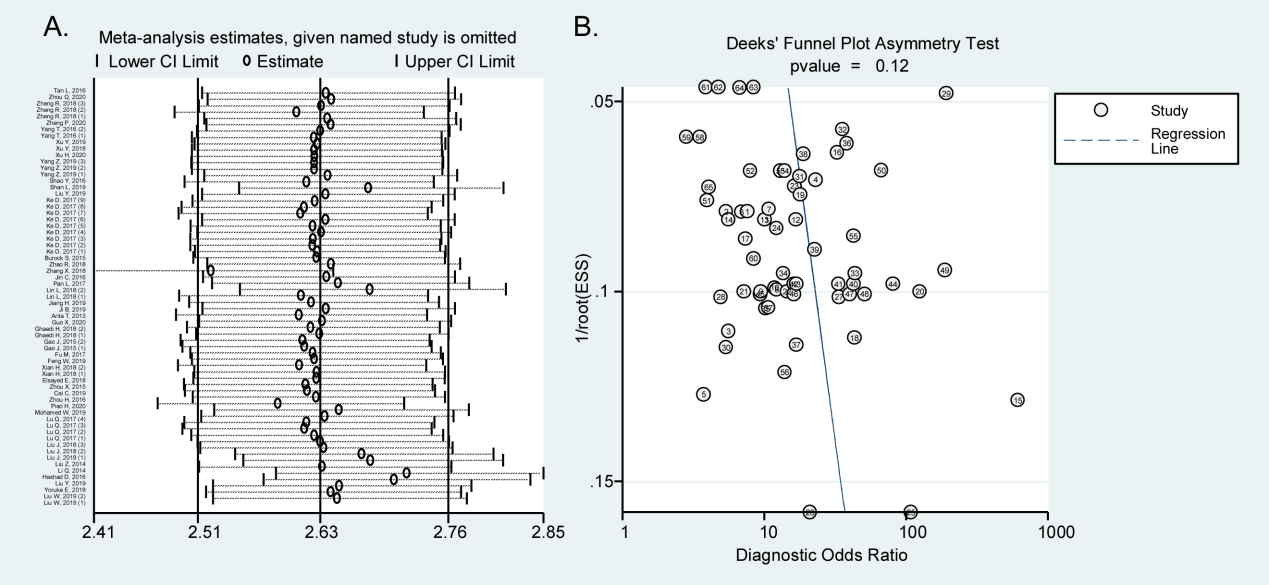
**

**Fig S1. Sensitivity analysis and publication bias. (A) Sensitivity analysis of the pooled studies. (B) Deeks’ funnel plot of the pooled studies.**
